# Supplementary material for: The Systems Biology Research Tool: evolvable open-source software
Source: BMC Syst Biol. 2008 Jun 29;2:55. doi: 10.1186/1752-0509-2-55 (PMC2446383; doi:10.1186/1752-0509-2-55)
Supplement: Additional file 1 — SBRT Archive. An archive of the current version of the Systems Biology Research Tool. [file 1752-0509-2-55-S1.zip › sbrt-1.4.0/doc/developers_guide/api/sbrt/kernel/math/HashLinearComb.Term.html]

HashLinearComb.Term


|  |  |  |
| --- | --- | --- |
| **Overview** \| **Package** \| **Class** \| **Use** \| **Tree** \| **Deprecated** \| **Index** \| **Help** | |  |
| **PREV CLASS**   **NEXT CLASS** | **FRAMES**    **NO FRAMES**     **All Classes** |  |
| SUMMARY: NESTED \| FIELD \| CONSTR \| METHOD | DETAIL: FIELD \| CONSTR \| METHOD |  |


---


## sbrt.kernel.math Class HashLinearComb.Term<V>

```
java.lang.Object
  sbrt.kernel.math.AbstractLinearComb.Term<V>
      sbrt.kernel.math.HashLinearComb.Term<V>
```

**Type Parameters:**: `V` - the variable type.

**All Implemented Interfaces:**: java.lang.Iterable<V>, LinearComb.Term<V>, Polynomial.Term<V>

**Enclosing class:**: HashLinearComb<V>

---

``` public static class HashLinearComb.Term<V> extends AbstractLinearComb.Term<V> ```

This class is a concrete implementation of `LinearComb.Term`.

**Author:**
:   This class was written and documented by Jeremiah Wright
    while in the Wagner lab.

---

**Constructor Summary**

`HashLinearComb.Term(double coefficient,
V variable)`
  
          Constructs a new linear combination term with the provided
coefficient and variable.

`HashLinearComb.Term(V variable)`
  
          Constructs a new linear combination term with the provided
variable.

`HashLinearComb.Term(V variable,
double coefficient)`
  
          Constructs a new linear combination term with the provided
coefficient and variable.


**Method Summary**

**Methods inherited from class sbrt.kernel.math.AbstractLinearComb.Term**

`equals, getCoefficient, getVariable, getVariables, hasAllVariables, hashCode, hasVariable, hasVariables, iterator, power, toString, variables`

**Methods inherited from class java.lang.Object**

`clone, finalize, getClass, notify, notifyAll, wait, wait, wait`

**Constructor Detail**

### HashLinearComb.Term

```
public HashLinearComb.Term(double coefficient,
                           V variable)
```

:   Constructs a new linear combination term with the provided
    coefficient and variable.

    **Parameters:**: `coefficient` - the coefficient of this term.: `variable` - the variable of this term.

---


### HashLinearComb.Term

```
public HashLinearComb.Term(V variable,
                           double coefficient)
```

:   Constructs a new linear combination term with the provided
    coefficient and variable.

    **Parameters:**: `variable` - the variable of this term.: `coefficient` - the coefficient of this term.

---


### HashLinearComb.Term

```
public HashLinearComb.Term(V variable)
```

:   Constructs a new linear combination term with the provided
    variable. The coefficient equals 1.

    **Parameters:**: `variable` - the variable of this term.


---


|  |  |  |
| --- | --- | --- |
| **Overview** \| **Package** \| **Class** \| **Use** \| **Tree** \| **Deprecated** \| **Index** \| **Help** | |  |
| **PREV CLASS**   **NEXT CLASS** | **FRAMES**    **NO FRAMES**     **All Classes** |  |
| SUMMARY: NESTED \| FIELD \| CONSTR \| METHOD | DETAIL: FIELD \| CONSTR \| METHOD |  |


---
